# A Diagnosis of Camptodactyly With Benign Joint Hypermobility Syndrome in a Patient Presenting With Fixed Flexion Deformity of the Fingers and Striae

**DOI:** 10.7759/cureus.26148

**Published:** 2022-06-21

**Authors:** Sekar Nishanth, Saravanamuttu Ushagowry

**Affiliations:** 1 Internal Medicine, Teaching Hospital Batticaloa, Batticaloa, LKA; 2 Rheumatologist, National Hospital of Sri Lanka, Colombo, LKA

**Keywords:** occupational therapy program, physiotherapy education, marfan disease, camptodactyly, generalized joint hypermobility

## Abstract

Camptodactyly is a genetic disorder that causes fixed flexion deformity of one or more fingers of single or both hands. It is very rare and the occurrence is very low amongst the children. It is linked to a handful of congenital connective tissue syndromes. It is passed onto generations with reduced expressivity. However, its association with benign joint hypermobility syndrome is rarely known. Joint hypermobility syndrome is a condition where there is extreme joint flexibility and it is related to a set of articular and extra-articular sequelae. We herein report a case of camptodactyly with benign joint hypermobility syndrome in a patient presenting with fixed flexion deformity of the fingers, joint hyperextensibility, and striae.

## Introduction

Camptodactyly is a fixed flexion deformity primarily involving the proximal interphalangeal joint [1]. Although it occurs sporadically, it may be inherited in the autosomal dominant manner and is very rarely seen in children (1%) [2]. It occurs mostly unilaterally (66%) and sometimes bilaterally (33%) in a symmetrical or asymmetrical fashion. The proposed pathophysiological basis is mainly the abnormal lumbrical muscle insertion. Also, the abnormal insertion of flexor digitorum superficialis and abnormal volar plate, and abnormal extensor hood are known to cause the deformity [2]. The diagnosis is made clinically. The radiology is routinely normal in the initial stages. Passive stretching and static splinting are the remedies to improve joint mobility. However, deformities that account for severe functional deprivation may require surgical techniques in the form of flexor digitorum superficialis tenotomy or corrective osteotomy [2].

Benson’s classification is used to type them according to the severity so that the management can be planned appropriately [2]. Type 1 basically includes those with isolated involvement of the little finger. Type 2 has abnormal lumbrical insertion or abnormal flexor digitorum superficialis origin. Type 3 has severe contractures with multiple digit involvement [2]. The clinical severity determines the treatment options. Camptodactyly also has a notable connection with several developmental dysmorphology syndromes including Marfan syndrome [2]. However, the association with benign joint hypermobility syndrome is seldom documented.

Benign joint hypermobility syndrome is a frequently missed rheumatological condition that causes highly flexible joints with associated pain [3]. The joints are easily movable beyond their range. Its prevalence around the globe is approximately 3% [3]. Many of them experience distressing symptoms such as pain and fatigue [4]. They are susceptible to developing significant musculoskeletal injuries [3]. Though it is speculated to be a part of hereditary diseases of connective tissue such as Marfan syndrome, Ehlers Danlos syndrome, and osteogenesis imperfecta, it is a milder, unique, and a commonly missed variant [5]. It is a grey area in the literature on how the benign joint hypermobility syndrome is related to and overlaps with the mild versions of hereditary diseases of connective tissue [5]. Benign joint hypermobility syndrome has a strong genetic association in the form of autosomal dominant inheritance [6]. Brighton has laid down the criteria for the diagnosis of joint hypermobility syndrome which considers the hyperextensibility of the elbows, knees, and fifth finger along with arthralgia lasting for three months or longer in four or more joints and several other aspects [6]. A multidisciplinary team approach is used for the management. Education, activity modification, and muscle strengthening exercises are the cornerstone of the treatment [6]. Open kinetic (distal extremity meets freely) and closed kinetic exercises (distal extremity moves against resistance) are used combinedly for strength training [6].

## Case presentation

A 15-year-old Sri Lankan boy was brought by his parents to the rheumatology clinic with fixed flexion deformities of the little fingers bilaterally with the associated diminished functional capacity of both hands and pain during the finger extensions. He had pain in all the five metacarpophalangeal and interphalangeal joints in both hands which had been persistent for more than nine months. No other joints were involved. He did not mention any persistent headaches, myalgia, and fatigue. There was no history of trauma or accidental fractures in the past. He denied any early morning joint stiffness or recent febrile episodes. He complained of an itchy rash on the dorsal aspect of the right index finger, which had been there for almost six months. He denied any altered bowel habits, urinary symptoms and blood, and mucus diarrhea. There were no features to suggest an autoimmune etiology. Neither did he have any family history of such deformity or rheumatoid arthritis. 

**Figure 1:**
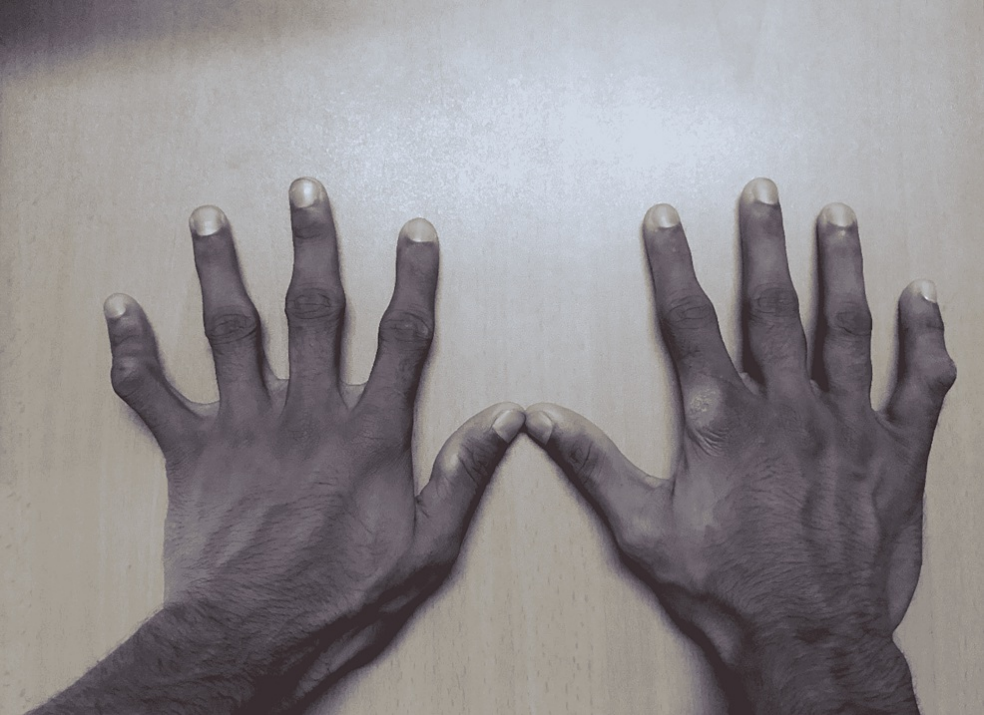
Fixed flexion deformity of both little fingers

On examination, he had a body mass index of 17.1 kg/m^2^. He was afebrile and not pale. He had no lymphadenopathy, clubbing or ankle edema. No visible ecchymotic patches on the skin were present. There were no tender points upon palpation. The fixed flexion deformity was observed on the proximal interphalangeal joints of both little fingers (Figure 1). There were no tender or swollen joints. He had a non-scaling eczematous rash on the dorsal aspect of the right index finger.

**Figure 2:**
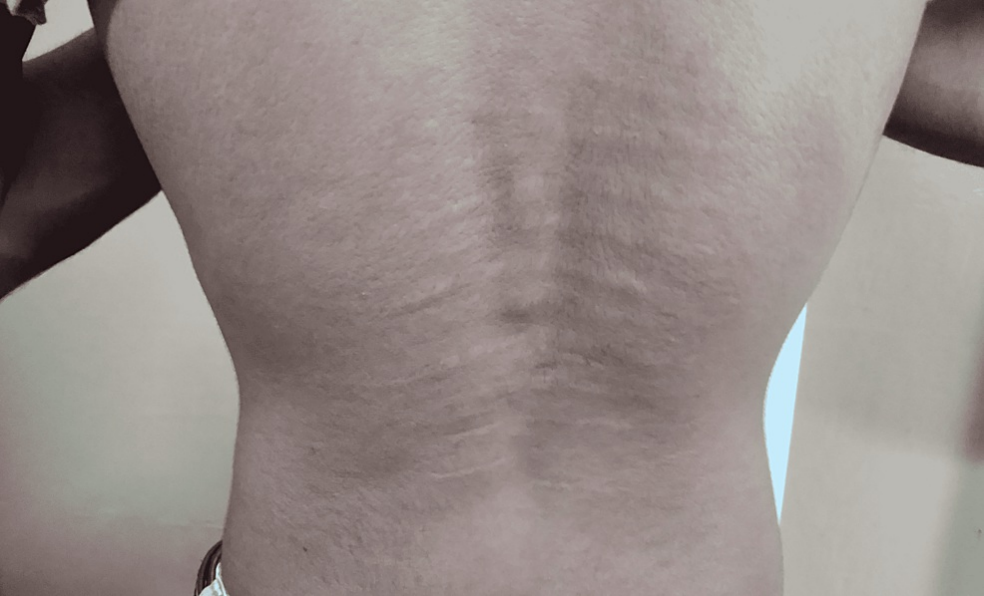
Striae atrophicae on the back

Prominent striae atrophicae was noticed on the back (Figure 2). But hernias or varicose veins were absent. The arm span to body height ratio was 0.8. Thump and wrist signs were absent. A high arched palate was also not observed.

**Figure 3:**
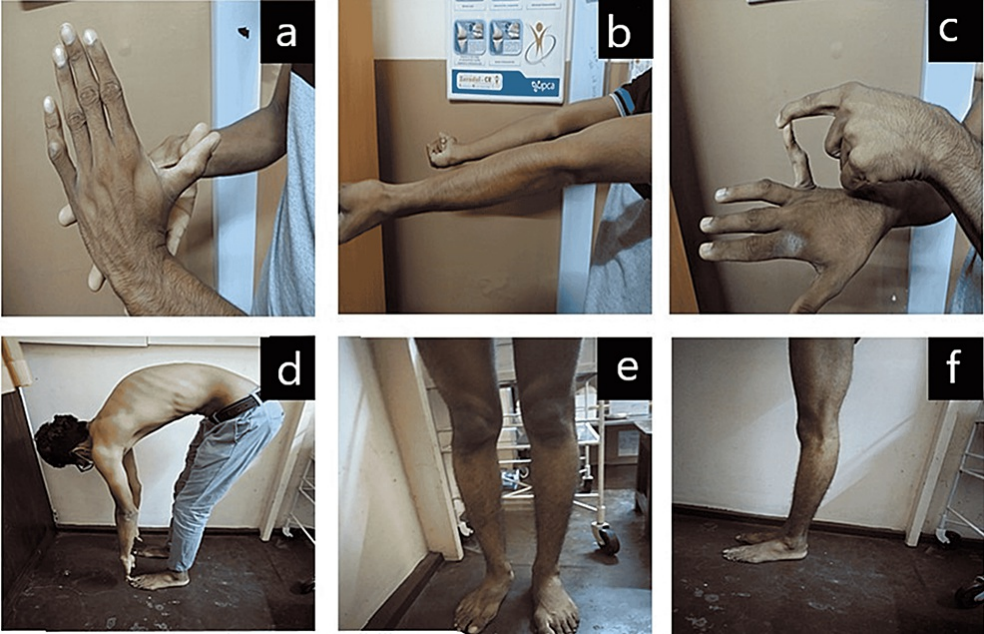
(a) Hyperextension of the thumb past 90 degrees at the metacarpophalangeal joint. (b) Hyperextension of the elbow beyond 10 degrees. (c) Hyperextension of the fifth metacarpophalangeal joint beyond 90 degrees. (d) Forward flexion with the hand touching the floor with the knee extension. (e) Anterior view of hyperextended knee beyond 10 degrees. (f) Lateral view of hyperextended knee beyond 10 degrees.

-Significant hyperextensibility was noted at the knees, elbows and 5th metacarpophalangeal joints (Figures 3a-3h). Modified Schober’s test was negative.

On fundoscopy examination, there was no ectopia lentils or any other abnormalies related to the connective tissue diseases. Cardiovascular examination revealed a pulse rate of 88 beats per minute which was of good volume and was regular. The cardiac apex was located at the fifth intercostal space in the midclavicular line and there was no detectable murmur. The respiratory, abdomen and neurology examinations were virtually unremarkable.

The following investigations were performed (Table 1).

**Table 1:** Set of investigations CRP: C-reactive protein, ESR: Erythrocyte sedimentation rate, CPK: Creatinine phosphokinase, ALT: Alanine transaminase, AST: Aspartate transaminase, ALP: Alkaline phosphatase, TSH: Thyroid stimulating hormone, UFR: Urine full report, ECG: Electrocardiogram

Test	Reference values	Results on admission
Full blood count		
White cell counts (10^3^/µL)	4-11	6.6
Neutrophils (10^3^/µL)	2-7	3.9
Lymphocytes (10^3^/µL)	1-5	2.1
Eosinophils (10^3^/µL)	< 0.5	0.3
Monocytes (10^3^/µL)	0.2-0.8	0.3
Platelets (10^3^/µL)	150-400	315
Hemoglobin (g/dL)	11-15	12.8
CRP (mg/L)	< 5	3
ESR (mm/hr)	< 22	5
CPK (µg/L)	10- 20	12
Rheumatoid factor (IU/mL)	< 14	5
Serum sodium^ (^mmol/L)	135-145	138
Serum potassium (mmol/L)	3.5-5.1	4.1
Serum calcium (mmol/L)	2.1-2.6	2.3
Serum Creatinine µmol/L	90-115	98
Blood urea (mmol/L)	3-7	3.8
ALT (U/L)	10-50	23
AST (U/L)	10-40	21
ALP (U/L)	25-150	142
Gamma- glutamyl transferase (U/L)	10-65	40
Total protein (g/L)	65-83	70
Serum albumin (g/L)	35-50	45
Serum globulin (g/L)	20-40	35
Total bilirubin (µmol/L)	5- 17	6
Serum 25 hydroxy cholecalciferol (ng/mL)	20-50	25
Free thyroxine (ng/dL)	1-1.7	1.1
TSH (mIU/ L)	10-28	12
Serum uric acid (mg/dL)	3.5-7	3.6
UFR: Pus cells	Nil	Nil
UFR: Red cells	Nil	Nil
UFR: Albumin	Nil	Nil
ECG		Sinus rhythm
2D echocardiography		Ejection fraction: 60% with normal valves Aortic root diameter: 21 mm
Ultrasound scan of the abdomen		No hepatosplenomegaly or lymphadenopathy
Chest x-ray		Normal

**Figure 4:**
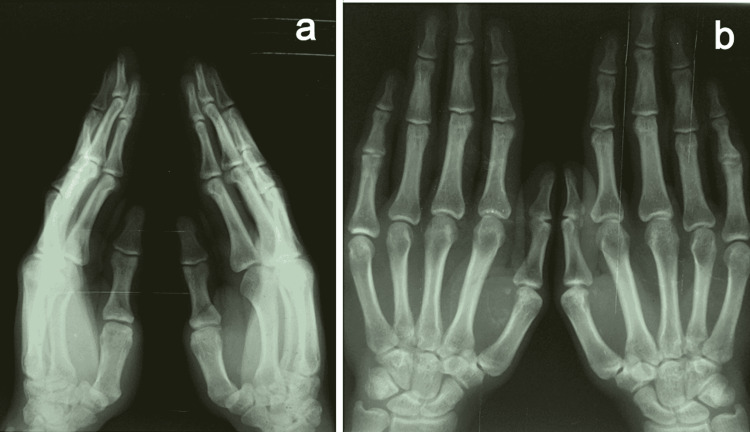
X-ray of the hand. (a) Lateral view. (b) Anteroposterior view.

The anteroposterior and lateral views of the hand x-ray (Figures 4a, 4b) were taken and were reported normal.

**Figure 5:**
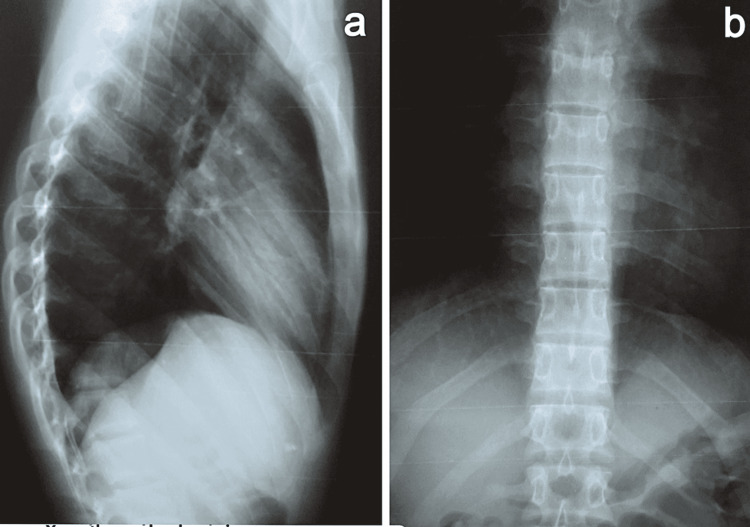
Thoracolumbar spine x-ray. (a) Lateral view. (b) Anteroposterior view.

The anteroposterior and lateral views of the thoracolumbar spine x-rays (Figures 5a, 5b) were taken which were normal. Ultrasound scan of the soft tissues of the hand excluded the synovitis. The patient and his parents were explained about the condition and its complications before proceeding to the next strategy. Analgesics were initiated for symptom relief.

The patient’s family was illustrated about the importance of involving the multidisciplinary team in the management of the patient. The consultant rheumatologist, physiotherapist, occupational therapist, podiatrist and consultant psychiatrist were included in the team of management. Physiotherapist opted to start the muscle strengthening exercises, proprioception training and postural alignments practices. Occupational therapists began pacing which is systematic graded approach to the participation in the activities. The activities to ensure sleep hygiene, relaxation and daily occupations were also bestowed upon the team. Podiatry team provided the advice regarding the appropriate foot wear and orthotics to support the patient achieve a higher functional independence. We also arranged counselling to the patient and his parents to ensure full participation of him in the activities of daily living and to gain a good motivation. Additionally, we made a dermatology referral regarding the eczematous non-scaly rash on dorsal aspect of the index finger which was diagnosed as psoriasiform eczema. He was offered topical corticosteroids for which he showed a good response. The wholesome approach helped to promote improved functional capacity and mental- wellbeing in the patient.

## Discussion

The diagnosis of benign joint hypermobility syndrome was made on the basis of the hyper flexibility at the elbow, finger metacarpophalangeal joints, knee, and thump. More sinister connective tissue diseases like Marfan’s syndrome and Ehlers Danlos syndrome were excluded clinically and with some investigations though some of the genetic tests were not available at the center. A five-point questionnaire was used to assess the severity [7]. The questionnaire along with the patient's responses are shown below (Table 2).

**Table 2:** The frequently used questionnaire with responses

The questions	Response	Points
• Do you consider yourself double-jointed?	Yes	1 point
• Can you now (or could you ever) place your hands flat on the floor without bending your knees?	Yes	1 point
• Can you now (or could you ever) bend your thumb to touch your forearm?	No	0 point
• As a child, did you amuse your friends by contorting your body into strange shapes or could you do the splits?	Yes	1 point
• As a child or teenager, did your shoulder or kneecap dislocate on more than one occasion?	No	0 Point
Total		3 points

He scored 3 points out of 5 which is more than 90% specific to benign joint hypermobility syndrome [7]. Several maneuvers were used to calculate the Beighton score for which the patient scored 6 (passive hyperextension of both elbows beyond 10 degrees, passive hyperextension of both knees greater than 10 degrees, and passive dorsiflexion of metacarpophalangeal joints up to 90 degrees on both sides) [7]. He fulfilled the revised Brighton’s criteria for benign joint hypermobility syndrome with one major criterion (polyarthralgia involving at least four joints lasting more than three months) and two minor criteria (Beighton score of 6 and skin changes such as striae) [8].

Apart, the patient’s fixed flexion deformity in both little fingers in the absence of any radiological abnormalities was diagnosed as camptodactyly. The association of camptodactyly with joint hypermobility syndrome is not well documented although it is related to congenital syndromes such as Marfan syndrome and Beal’s syndrome [9].

Benign joint hypermobility syndrome is an overlooked entity that causes chronic pain and fatigue. It is sometimes missed due to the variable nature of the presentations. However, the affected patients are liable to develop fatigue, headache, orthostatic hypotension, anxiety, hernias, functional gastrointestinal disturbances, vasovagal syncope, dysautonomia, and genitourinary symptoms [10]. Therefore, it’s necessary to obtain a comprehensive history and perform a detailed clinical examination. After excluding the more alarming heritable connective tissue diseases, a thorough clinical examination of the musculoskeletal system helps to establish a unifying diagnosis and to assess the extent of burden.

Collaborative care from a multidisciplinary team of rheumatologists, physiotherapists, occupational therapists, podiatrists, psychologists, and orthopedic surgeons is important for a successful treatment outcome. The probable reassurance is that joint laxity may decline with advancing age so the symptoms will gradually improve.

The prime goal of physiotherapy is to tackle muscle inhibition and build up muscle strength. The muscle strengthening exercises, active mobilization exercises, and proprioception exercises are included. Once the neutral resting position of the joint is achieved, retraining is done to gain dynamic control whilst moving the adjacent joints [11].

Occupational therapists assess the patient with the view of increasing sleep, relaxation, and activities to ameliorate the functionality. For example, they are provided with specialized pens/pencil grips, finger splints, and angled desktops in order to improve their quality of life and performance [11].

Podiatrists help to wear the appropriate supportive footwear and orthotics to stabilize the feet and other muscle groups [12]. The psychotherapists can offer counseling to boost confidence and motivation which ensures good treatment compliance. Due to the uniplanar and supported weighting bearing nature, some activities involving side-to-side movements such as swimming, rowing, and cycling are highly encouraged [13].

This is the very first case report around the world to underline the association of camptodactyly with benign joint hypermobility syndrome. However, one case report from Southern Turkey describes three instances of camptodactyly - arthropathy - coxa vara- pericarditis syndrome (genetic mutation in the proteoglycan 4 gene), which mimicked juvenile idiopathic arthritis and were inappropriately treated [14]. Similarly, a case of Blau syndrome (mutation in nucleotide-binding oligomerization domain-containing two genes) was reported from Palestine with camptodactyly and bilateral intermediate uveitis and responded well to subcutaneous Adalimumab, biologic disease-modifying agents [15].

The criterion for the selection of candidates for therapy is unclear. There was a reported case of radiographic remodeling of the proximal phalangeal head using stretching exercise in patients with camptodactyly [16]. It concluded that graded stretching exercises helped to restore mobility of the proximal interphalangeal joints and gradually brought back proximal phalangeal head roundness and concentricity in those with infantile-type camptodactyly. Some case reports linked the camptodactyly to the anomalous origin of the flexor digitorum superficialis tendon [17]. The general consensus regarding the interventions was that the surgical correction is certainly assigned to those who have preoperative proximal interphalangeal joint contracture of more than 60 degrees [18].

Nonopioid analgesic care is provided to relieve symptoms. Prolotherapy is sometimes used for the management of joint hypermobility syndrome. Irritants such as dextrose solution are injected into the joints to mount a short-lasting inflammatory reaction, which initiates a reparative cascade to produce more extracellular matrix and collagen, giving additional strength and withstanding ability [19]. It also slows down the degenerative changes due to joint hypermobility, thereby postponing the development of precocious osteoarthritis [19]. A current shift from active to passive care for patients with hypermobility syndrome has proven to be so much beneficial to the affected patients.

## Conclusions

This is the world’s first reported case of benign joint hypermobility syndrome with associated camptodactyly, which is a rarely fixed flexion deformity of the fingers. Hypermobility syndrome, if left unaddressed, can result in so many complications including chronic pain, musculoskeletal injuries, and fatigue. Therefore, it requires a holistic approach with the inclusion of rheumatologists, physiotherapists, occupational therapists, podiatrists, and psychotherapists. Camptodactyly, on the other hand, is approached with graded stretching exercises and if markedly severe, necessitates surgical correction for a better functional outcome.
